# Magnetic Resonance Imaging and 99Tc WBC-SPECT/CT Scanning in Differential Diagnosis between Osteomyelitis and Charcot Neuroarthropathy: A Case Series

**DOI:** 10.3390/tomography10080098

**Published:** 2024-08-22

**Authors:** Sara Cecchini, Cristina Gatti, Daniela Fornarelli, Lorenzo Fantechi, Cinzia Romagnolo, Elena Tortato, Anna Rita Bonfigli, Roberta Galeazzi, Fabiola Olivieri, Giuseppe Bronte, Enrico Paci

**Affiliations:** 1 Department of Radiology, IRCCS INRCA, 60127 Ancona, Italy; s.cecchini@inrca.it (S.C.); e.paci@inrca.it (E.P.); 2 Diabetic Foot Clinics, IRCCS INRCA, 60127 Ancona, Italy; c.gatti@inrca.it (C.G.); e.tortato@inrca.it (E.T.); 3 Unit of Nuclear Medicine, IRCCS INRCA, 60127 Ancona, Italy; d.fornarelli@inrca.it (D.F.); l.fantechi@inrca.it (L.F.); 4 Department of Nuclear Medicine, “Ospedali Riuniti” Hospital, 60126 Ancona, Italy; cinzia.romagnolo@ospedaliriuniti.marche.it; 5 Scientific Direction, IRCCS INRCA, 60127 Ancona, Italy; a.bonfigli@inrca.it (A.R.B.); f.olivieri@univpm.it (F.O.); 6 Clinic of Laboratory and Precision Medicine, IRCCS INRCA, 60127 Ancona, Italy; r.galeazzi@inrca.it; 7 Department of Clinical and Molecular Sciences (DISCLIMO), Università Politecnica delle Marche, 60126 Ancona, Italy

**Keywords:** Charcot neuroarthropathy, osteomyelitis, diabetes, magnetic resonance imaging, 99mTc-HMPAO–WBC SPECT/CT

## Abstract

Background: Distinguishing between Charcot Neuroarthropathy (CN), osteomyelitis (OM), and CN complicated with superimposed OM in diabetic patients is crucial for the treatment choice. Given that current diagnostic methods lack specificity, advanced techniques, e.g., magnetic resonance imaging (MRI) and 99mTc-HMPAO–WBC Single Photon Emission Computed Tomography (SPECT/CT), are needed. This study addresses the challenges in distinguishing OM and CN. Methods: We included diabetic patients with CN and soft tissue ulceration. MRI and 99mTc-HMPAO–WBC SPECT/CT were used for the diagnosis. The patients were classified into three probability levels for OM (i.e., Definite, Probable, and Unlikely) according to the Consensus Criteria for Diabetic Foot Osteomyelitis (CC-DFO). Results: Eight patients met the eligibility criteria. MRI, supported by SPECT-CT and CC-DFO, showed consistency with the OM diagnosis in three cases. The key diagnostic features included the location of signal abnormalities and secondary features such as skin ulcers, sinus tracts, and abscesses. Notably, cases with inconclusive MRI were clarified by SPECT/CT, emphasizing its efficacy in challenging scenarios. Conclusions: The primary objective of this study was to compare the results of MRI and 99mTc-HMPAO–WBC SPECT/CT with the CC-DFO score in the diabetic foot with CN and suspected OM. Advanced imaging offers a complementary approach to distinguish between CN and OM. This can help delineate the limits of the disease for presurgical planning. While MRI is valuable, 99mTc-HMPAO–WBC SPECT/CT provides additional clarity, especially in challenging cases or when metallic implants affect MRI accuracy.

## 1. Introduction

The diagnosis and treatment of foot-related diseases in diabetic patients pose challenges, especially in distinguishing Charcot neuroarthropathy (CN) from osteomyelitis (OM) [1,2]. The prevalence of CN in diabetic individuals ranges from 0.08% to 7.5%, reaching up to 13% in high-risk patients. CN is a non-infectious condition in which peripheral neuropathy plays a crucial role in reducing pain sensation and proprioception. While the exact mechanism is not fully understood, it is acknowledged that inflammation pathways contribute to the development of the condition, leading to prolonged foot inflammation. In the natural history of Charcot neuroarthropathy, inflammation in the active phase is followed by variable degrees of destruction of the skeletal architecture, with deformity and instability of the foot and the ankle. In the majority of cases, the condition eventually reaches a stable state, even though the structural damage remains permanent. However, there is a risk of re-activation of a Chronic Charcot foot in about 23% of cases within a mean interval of 27 months [1].

CN symptoms mimic OM and can be complicated with OM [3]. Distinguishing between CN and OM is crucial due to distinct treatment strategies. In challenging cases involving diabetic foot with ulceration, there is not a single definitive method to differentiate between CN and OM [3,4]. Bone biopsy and tissue cultures are commonly regarded as the ‘gold standard’ for diagnosing OM. However, limitations such as their invasiveness, cost, unstable joints or active infections in patients with diabetes-related white cell function impairment make these tests rarely used in clinical practice [5,6]. Clinical examinations and inflammatory markers, especially probe-to-bone tests and measurement of erythrocyte sedimentation rate or better C-reactive protein, may be helpful but all are relatively non-specific [7,8]. Imaging tests generally begin with plain X-rays [9]. When these are inconclusive or more detail of bone or soft tissue abnormalities is required, advanced imaging techniques are needed [10,11].

Magnetic resonance imaging (MRI) is widely considered the best available radiological imaging technique for evaluating both soft tissues and bony structures, with an overall sensitivity of 90% and a specificity of 80% in diagnosing diabetic foot osteomyelitis (DFO) [12,13]. Nuclear medicine, specifically radiolabeled white blood cell scintigraphy, provides specificity in detecting bone infections, and the relatively newer SPECT/CT hybrid imaging modality holds promise in accurately discerning between OM and soft tissue infections [14,15]. Diagnosing DFO requires a systematic approach that includes clinical, imaging, microbiological, and histopathological methods. To guide OM diagnosis in the diabetic foot, an international panel of experts proposed consensus criteria for diagnosing DFO (Consensus Criteria in Diabetic Foot Osteomyelitis: CC-DFO) with a novel approach that combines a variety of clinical, laboratory, and imaging studies to suggest the probability of its diagnosis [2]. In the proposed scheme assessing the probability of DFO, both MRI and WBC scans are included in the imaging studies relevant to the CC-DFO criteria. 

In particular, the expert panel considers MRI highly effective for diagnosing DFO, especially when utilizing fluid-sensitive and fat-suppressed sequences. MRI is specifically mentioned in the criteria for “Definite” diagnosis, where an intraosseous abscess found on MRI contributes to a definitive diagnosis of osteomyelitis. Additionally, MRI is mentioned in the criteria for “Probable” diagnosis, where MRI showing bone oedema with other signs of osteomyelitis (such as disappearance of bony contours and presence of soft tissue changes near the bone abnormality) supports a probable diagnosis. 

On the other hand, the panel of experts identifies the WBC scan as the method with the highest specificity in diagnosing DFO, as it can detect leukocytic infiltration. Indeed, radiolabeled white blood cells (WBC) remain the top choice for nuclear imaging in musculoskeletal infections. Labeling with 99mTechnetium is favored over 111Indium due to superior radiation properties, lower radiation dose, enhanced image resolution, and reduced expenses. This method excels in detecting leukocytic infiltration and is unaffected by recent or ongoing antibiotic treatment. It reliably identifies both acute and chronic infections, including low-grade cases.

## 2. Materials and Methods

This is a single-center observational pilot study aimed at evaluating MRI and SPECT-CT with labeled leukocytes in relationship with the score assessing the probability of osteomyelitis in the diabetic foot. Thus, patients with CN, for whom additional OM was suspected, underwent MRI and 99mTc-HMPAO–WBC SPECT/CT for the final confirmation of this suspicion.

Patients were enrolled and clinically evaluated at the Diabetic Foot Clinics, IRCCS INRCA, Ancona, Italy. Imaging was performed at the Department of Radiology, Unit of Nuclear Medicine, of the same hospital.

Both diagnostic methods (MRI and 99mTc-HMPAO–WBC SPECT/CT) were employed to distinguish between CN and OM, aiming to provide valuable guidance for management and early decision-making. The primary objective was the comparison of the results of each method (MRI and scintigraphy with labeled leukocytes with the SPECT/CT technique, respectively) with the CC-DFO score, in the diabetic foot with Charcot neuropathic osteoarthropathy and suspected osteomyelitis. The criteria for the final diagnosis were based on clinical follow-up, specifically the response to targeted therapy for Charcot and/or osteomyelitis. 

The inclusion criteria were as follows: (1) diagnosis of type 1 or 2 diabetes; (2) clinical suspicion of OM in diabetic foot with existing CN (CN in the acute phase or previous CN flare-up); and (3) signature of written informed consent. The exclusion criteria were as follows: (1) ulcerated diabetic foot in the absence of the clinical criteria of CN and/or suspected superimposed OM; (2) critical ischemia (TCPO2 < 30 mmHg); (3) inability to perform MRI (patients with a non-MRI compatible cardiac pacemaker, splinters or metal fragments in the ocular, visceral or intracranial area, catheters, intracorporeal electrodes, neurostimulators, vascular filters, stents and metal coils whose characteristics are not MRI safe); and (4) lack of written informed consent. Eligible patients underwent X-ray and MRI of the foot and scintigraphy with radiolabeled leukocytes with the SPECT/CT technique. The final diagnosis of OM was formulated using probabilistic criteria with the integration of clinical–laboratory and instrumental data according to the diagnostic categories expressed in the CC-DFO.

The data were collected from the patients’ medical records and the outcomes of the MRI and scintigraphy reports with radiolabeled leukocytes with the SPECT/CT technique. The data were entered anonymously into a specific database, attributing to each patient only a numerical code and the date of the investigations carried out. The data collected were age, gender, ulcer location, clinical examination of the foot, blood chemistry tests, date and findings of the conventional x-ray investigation of the foot, date and findings of the MRI investigation, and date and findings of 99mTc-HMPAO–WBC SPECT/CT. 

For 99mTc-HMPAO–WBC SPECT/CT, the acquisition and interpretation protocol followed the European Association of Nuclear Medicine (EANM) procedural guidelines. Based on the consensus criteria for diagnosing OM in the diabetic foot (CC-DFO) proposed by Berendt et al. [3], the patients were classified into three probability categories for OM (Definite, Probable, Unlikely).

## 3. Results

Among the diabetic subjects evaluated at the outpatient clinics in the last 3 years, only eight consecutive patients with CN complicated by soft tissue ulceration met the eligibility criteria. These patients presented symptoms resembling CN, characterized by a warm, swollen, painful, and erythematous foot with superimposed soft tissue ulceration, raising suspicion of concurrent OM.

Table S1 reports the clinical presentation, radiographic findings, MRI, and 99mTc-HMPAO–WBC SPECT/CT results, along with OM probability categories based on CC-DFO for eight cases involving Charcot neuropathy with a concomitant foot ulcer.

**Figure 1:**
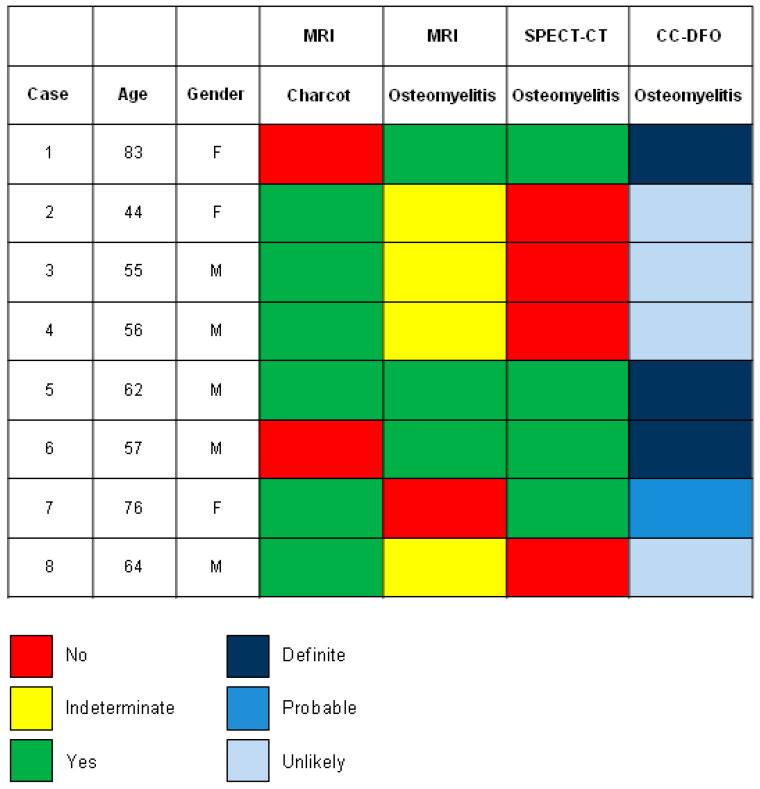
Classification of each patient’s MRI diagnosis for both CN and OM, SPECT-CT diagnosis for OM, and CC-DFO diagnostic criteria for OM.

In our series of eight consecutive patients with clinical and/or radiographic evidence of CN complicated by clinical suspicion of superimposed OM, the final diagnosis was CN complicated by osteomyelitis in two cases (case 5 and case 7; Figure 1), CN in four cases (cases 2, 3, 4 and 8; Figure 1) and OM on its own in two cases (cases 1 and 6; Figure 1).

The MRI was consistent with OM in three out of four cases of OM (75%), confirmed by SPECT-CT and CC-DFO (cases 1, 5, and 6; Figure 1). In these cases, the location of the signal abnormality (hyperintensity of bone marrow on T2-weighted images with marked hypointensity on T1-weighted images associated with cortical disruption involving weight-bearing surface) and soft tissue findings (adjacent sinus tract, fluid collection, and soft tissue mass) represented key features for a correct diagnosis. 99mTc-HMPAO–WBC SPECT/CT confirmed the localization of the infective foci into the bone and soft tissue with an excellent definition of the extent of the process (Figure S1). In the single case in which the MRI showed a negative result for OM, whereas the SPECT-CT demonstrated the presence of osteomyelitis, metallic osteosynthesis implants were present, which limited a comprehensive bone assessment (case 7, Table S1). As is well-known, one of the primary limitations affecting the diagnostic accuracy of MRI is the occurrence of ferromagnetic artifacts, due to the presence of metallic synthesis devices.

In the four cases in which the MRI yielded inconclusive results (indeterminate results: neither positive nor negative for OM), the bone marrow edema was the only finding of the MRI (cases 2, 3, 4, and 8; Figure 1 and Table S1). In these cases, SPECT-CT ruled out the presence of OM according to the CC-DFO and correctly localized infective foci into soft tissue (Figure S2).

In our study, MRI confirmed clinical and/or radiographic suspicion of Charcot CN in six out of eight cases (Table S1). Specifically, in three cases, MRI revealed bone marrow edema with subchondral and periarticular distribution, along with edema in soft tissues and muscles consistent with early active Charcot foot (cases 2, 5, and 7, Table S1). In one case, MRI displayed extensive bone marrow edema, cartilage erosion, subchondral bone fragmentation, joint subluxation/dislocation, and debris formation compatible with active middle-stage Charcot foot (Fragmentation stage; case 3, Table S1). Finally, in two cases, MRI demonstrated end-stage Charcot foot (Chronic Charcot foot) with fusion and consolidation of large fragments, deformities, and mild edema of the midfoot and hindfoot due to re-activation (cases 4 and 8, Table S1). Intraoperative bone biopsies and cultures are the gold standard and the definitive tests for diagnosing osteomyelitis. However, in our series, except for one case where a bone biopsy was possible (case 6; Figure 1), unstable joints or active infections prevented its execution. Consequently, the criteria for the final diagnosis were based on clinical follow-up, specifically the response to targeted therapy for Charcot and/or osteomyelitis. Patients diagnosed with osteomyelitis according to the probabilistic criteria of CC-DFO showed improvement after antibiotics and/or removal of the infected bone. Conversely, all patients classified as unlikely to have osteomyelitis resolved the fragmentations with immobilization and offloading without requiring antibiotics or surgery. In these cases, ulcerations healed with wound debridement.

## 4. Discussion

In our series of eight consecutive patients with clinical suspicion of CN complicated by superimposed OM, MRI yielded conclusive results for OM that were consistent with 99mTc-HMPAO–WBC SPECT/CT in all three patients classified into the Definite category based on the consensus criteria for diagnosing OM in the diabetic foot (CC-DFO). In these cases, the location and distribution of bone marrow edema with focal involvement affecting the weight-bearing surface of the bone, the poor definition of the margins of a bone on T1-weighted images, and the presence of secondary features such as skin ulcers, sinus tracts and abscesses, improve the diagnostic accuracy of MRI.

Diagnosing CN is relatively straightforward in patients without foot ulceration. The challenge for clinicians arises when dealing with diabetic patients who present with a hot, swollen, painful, and erythematous foot with superimposed soft tissue ulceration. Distinguishing between these two categories is crucial, as the approach to treatment varies significantly depending on the disease. DFO typically necessitates antibiotic therapy and surgical intervention, while Charcot disease requires proper offloading, occasionally followed by surgical correction [16].

Imaging plays a pivotal role in differentiating between CN and OM and can provide valuable guidance for management and early decision-making regarding the need for amputation [17]. After initial radiography, MRI with fluid-sensitive and fat-suppressed sequences shows an overall sensitivity of 90% and a specificity of 80% in the diagnosis of OM [13,18,19]. Nevertheless, the accuracy of MRI for the detection of OM complicating CN has not been reported in the literature.

As evidenced in the literature, the location of the signal abnormality and soft tissue findings may be the only key features for differential diagnosis [20]. OM occurs almost exclusively by the contiguous spread of infection to the bone from adjacent skin ulceration. CN is not related to an overlying skin ulcer, usually involves multiple midfoot bones and shows marrow abnormality in the periarticular and subchondral distribution [21].

Diagnosing OM complicating CN can be clinically challenging. MRI is the most effective imaging modality for diagnosing this complication. The “ghost sign”, seen as poorly defined bone margins on T1-weighted images that become clearer after contrast administration or on T2-weighted images, is particularly useful in identifying superimposed infection [22].

The mere presence of bone marrow edema is present similarly on both active CN and OM and is a very non-specific finding in both conditions, which is one of the main reasons for the limited specificity of MRI [23]. In this scenario, the additional contribution of 99mTc-HMPAO–WBC SPECT/CT was to rule out the presence of OM and better define the localization of the infection outside the bone and in the soft tissue [24,25]. In particular, in our series, cases classified as “indeterminate” on MRI (cases 2, 3, 4, and 8; Figure 1) but later excluded for OM by SPECT/CT showed clinical deformity and collapse of the midfoot with pressure ulceration. On MRI, they displayed a pattern of bone marrow edema that was generally diffuse, periarticular, and subchondral, but also focal, involving a single bone on weight-bearing surfaces. This was accompanied by disorganization and fragmentation of the bone and a profound low signal on T1-weighted sequences resembling infection superimposed upon CN, without, however, loss of definition of the bone margins. Therefore, the MRI findings could suggest OM complicating CN but do not definitively confirm it as there were no accompanying soft tissue changes such as sinus tracts or abscesses near the bone abnormality. In these cases, where MRI findings alone were insufficient to confirm or rule out OM, SPECT/CT provided additional information, confirming the absence of OM and documenting soft tissue infection, thereby facilitating accurate diagnosis and treatment planning. SPECT/CT offers numerous advantages over standard or dual isotope scans: superior cortical spatial resolution, cost-effectiveness compared to dual scintigraphy, reduced radiation exposure, and compatibility with various isotope agents. In WBC scintigraphy, integrating SPECT/CT with early images (3–4 h post-injection) enhances the precision of infection site localization and assessment of infection extent. This integration also improves the differentiation between soft tissue infection and osteomyelitis, thereby enhancing diagnostic accuracy [26,27,28].

## 5. Conclusions

Our cases highlight the need for labeled WBCs with SPECT/ CT in patients in whom the diagnosis remains uncertain on MRI or when osteosynthesis material is present at the site of interest because 99mTc-SPECT/CT can provide excellent demarcation of necrotic from viable tissues and can delineate the extent of infection. The information obtained from such precise findings is helpful for tissue-sparing presurgical planning, making 99mTc-SPECT/CT clinically useful and cost-effective.

The selection of the most suitable advanced imaging modality requires consideration of the diagnostic utility, equipment availability, costs, waiting times, contraindications, and patient preferences. Due to the complexity and lack of consensus, standardized diagnostic methods and evidence-based approaches are crucial. It is necessary to conduct large-scale studies comparing MRI and advanced nuclear techniques for specific populations with suspected Charcot foot and OM to determine the most suitable imaging tool and assess cost-effectiveness.

## Data Availability

Data are contained within the article.

## Supplementary Materials

The following supporting information can be downloaded at: https://www.mdpi.com/article/10.3390/tomography10080098/s1. Table S1: Clinical and radiological features for each patient and relative outcomes. Figure S1: MRI and 99mTc-HMPAO-WBC SPECT-CT scan of case 1. Figure S2: MRI and 99mTc-HMPAO-WBC SPECT-CT scan of case 2. Figure S3: MRI and 99mTc-HMPAO-WBC SPECT-CT scan of case 3. Figure S4: MRI and 99mTc-HMPAO-WBC SPECT-CT scan of case 4. Figure S5: MRI and 99mTc-HMPAO-WBC SPECT-CT scan of case 5. Figure S6: MRI and 99mTc-HMPAO-WBC SPECT-CT scan of case 6. Figure S7: MRI and 99mTc-HMPAO-WBC SPECT-CT scan of case 7. Figure S8: MRI and 99mTc-HMPAO-WBC SPECT-CT scan of case 8.
