# Supplementary material for: Magnetic Resonance Imaging and 99Tc WBC-SPECT/CT Scanning in Differential Diagnosis between Osteomyelitis and Charcot Neuroarthropathy: A Case Series
Source: Tomography. 2024 Aug 22;10(8):1312–9. doi: 10.3390/tomography10080098 (PMC11359419; doi:10.3390/tomography10080098)
Supplement: Supplementary file 1 [file tomography-10-00098-s001.zip › Figure S1_Case 1.pdf]

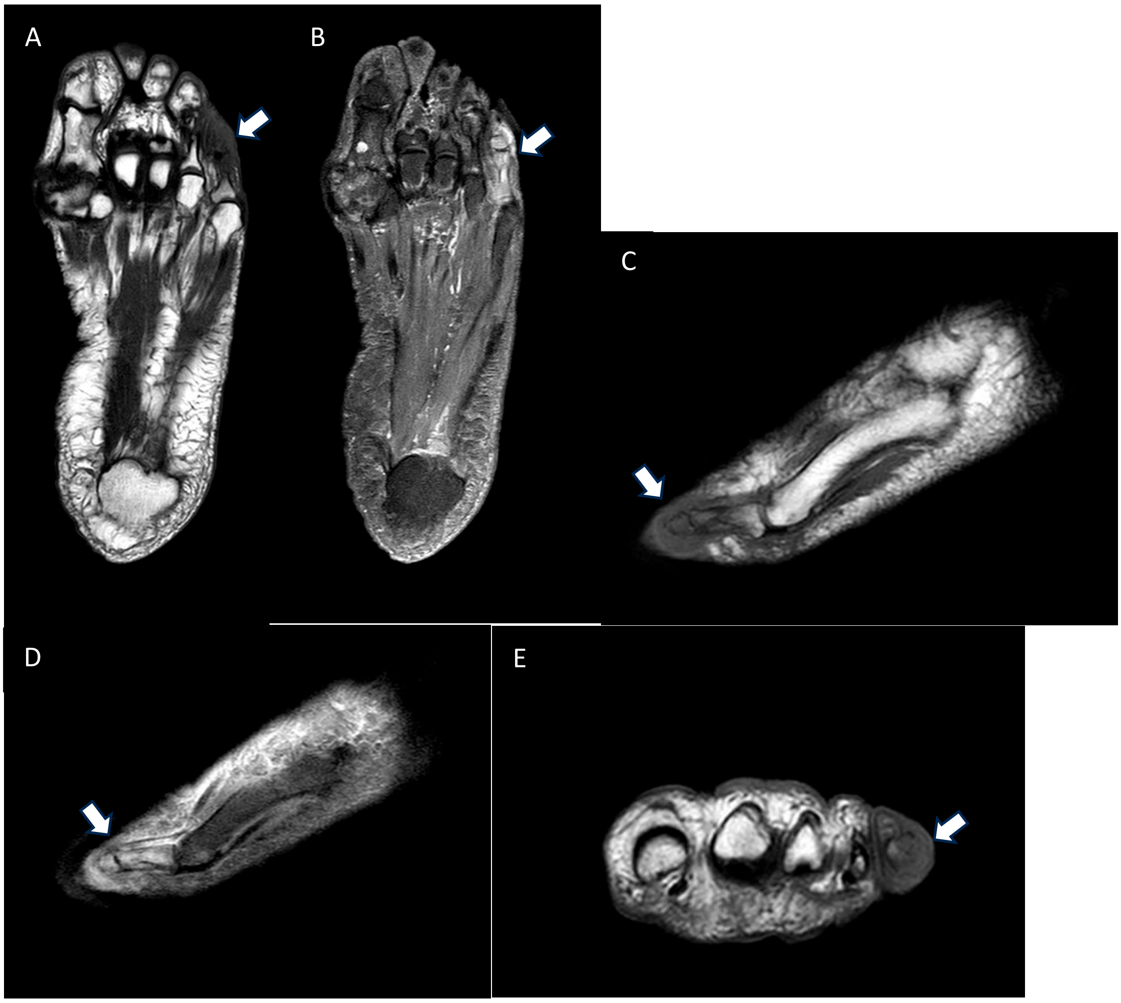
A
B
C
D
E

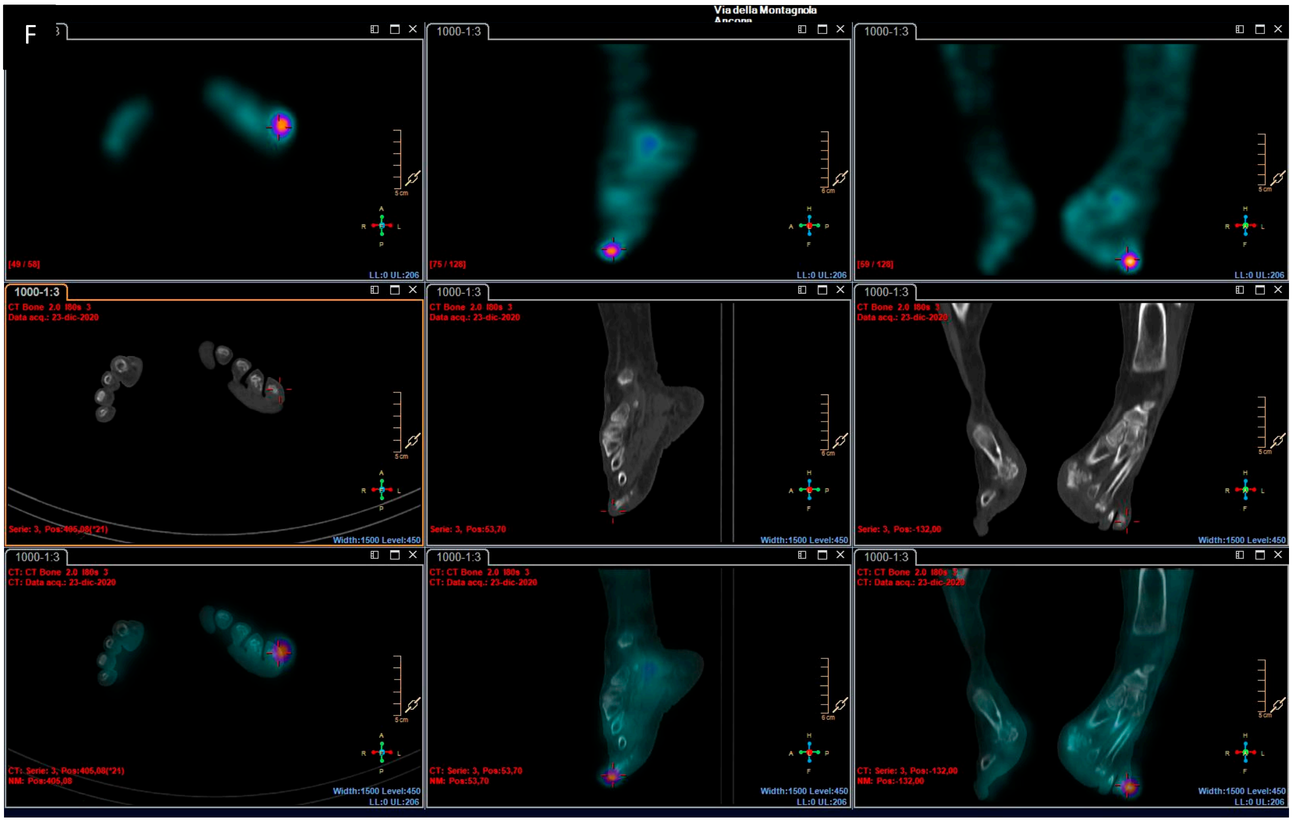
F
Via della Montagnola
1000-1:3
CT Bone 2.0 I80s 3
Data acq.: 23-dic-2020
Width:1500 Level:450
LL:0 UL:206

Supplementary Figure S1.

Case 1: 83-year-old female with diabetes mellitus and peripheral neuropathy with 3 weeks of foot pain, swelling and erythema. She also presents an ulceration on the lateral aspect of her fifth foot, positive to probe to bone test. The question from the clinician is if there is an osteomyelitis or a Charcot foot.

On MRI there are not changes consist with Charcot neuropathy. MRI shows a profound low signal on T1-weighted sequence with loss of definition of the bone margins at the proximal, middle and distal phalanx of the fifth toe, suggestive of osteomyelitis (A, B, C, D, E).

99mTc-HMPAO-WBC scan shows increase in uptake in time located at the proximal, middle and distal phalanx of the fifth toe on the lateral profile consistent with osteomyelitis (F).

Final diagnosis: osteomyelitis.
