# Supplementary material for: Magnetic Resonance Imaging and 99Tc WBC-SPECT/CT Scanning in Differential Diagnosis between Osteomyelitis and Charcot Neuroarthropathy: A Case Series
Source: Tomography. 2024 Aug 22;10(8):1312–9. doi: 10.3390/tomography10080098 (PMC11359419; doi:10.3390/tomography10080098)
Supplement: Supplementary file 1 [file tomography-10-00098-s001.zip › Figure S2_Case2.pdf]

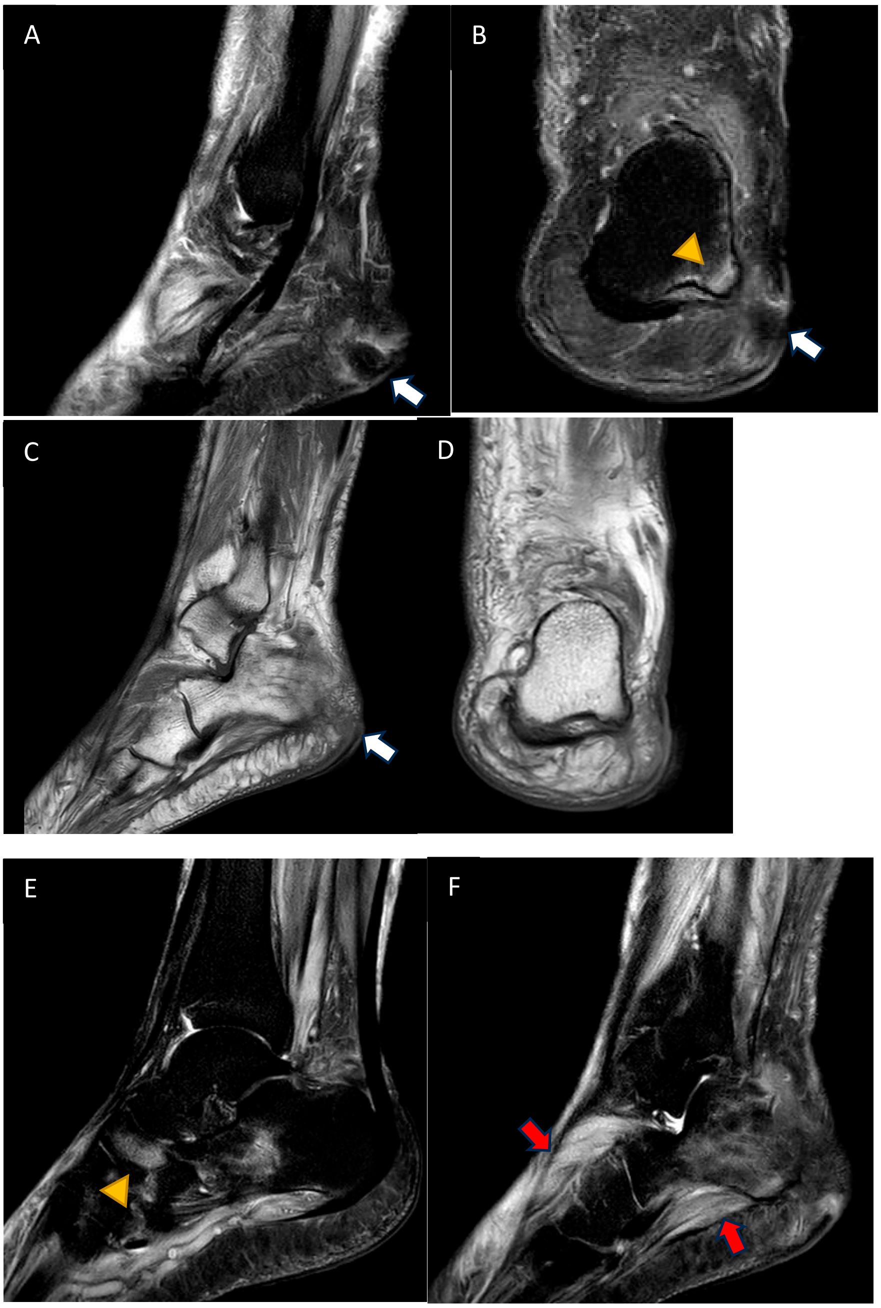
A
B
C
D
E
F

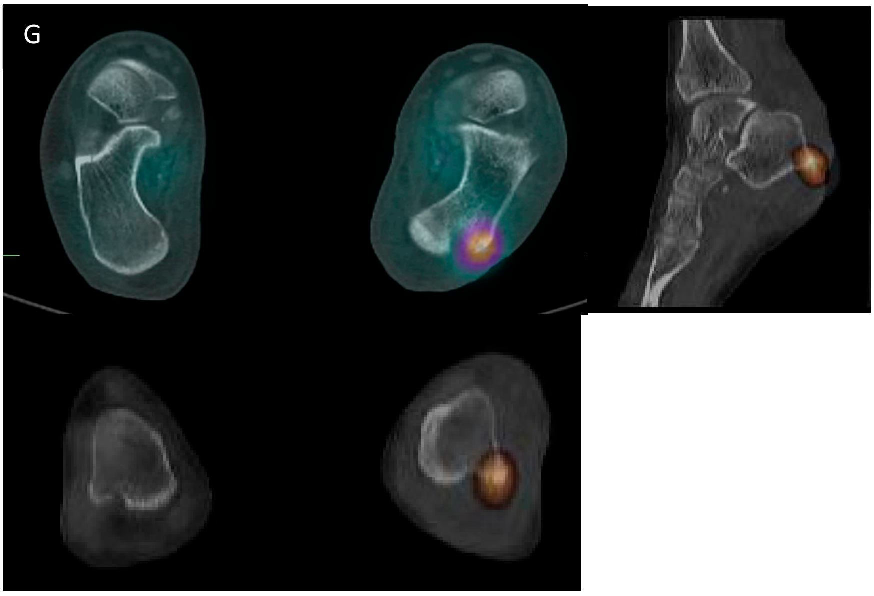

Supplementary Figure S2.

Case 2: a 44-year-old female with swollen, warm foot and ulceration on the lateral calcaneal site.

MRI: fluid sensitive sequence (Short tau inversion recovery; STIR) demonstrates skin ulceration on the lateral calcaneal site and bone marrow edema within the calcaneus (A, B); T1-weighted sequence doesn't show focal replacement of fatty bone marrow signal within the calcaneus bone with regular bony contours (C, D). There was also bone marrow edema in navicular bone and diffuse soft tissue edema possible manifestation of active Charcot disease (E, F). It could be Charcot with possible osteomyelitis.

Tc-HMPAO-WBC SPECT-CT: The result of SPECT-CT suggests a soft tissue inflammation on the inferolateral surface of the heel without evidence of osteomyelitis (G).

Final diagnosis: Charcot without superimposed osteomyelitis.
