# Supplementary material for: Magnetic Resonance Imaging and 99Tc WBC-SPECT/CT Scanning in Differential Diagnosis between Osteomyelitis and Charcot Neuroarthropathy: A Case Series
Source: Tomography. 2024 Aug 22;10(8):1312–9. doi: 10.3390/tomography10080098 (PMC11359419; doi:10.3390/tomography10080098)
Supplement: Supplementary file 1 [file tomography-10-00098-s001.zip › Figure S3_Case 3.pdf]

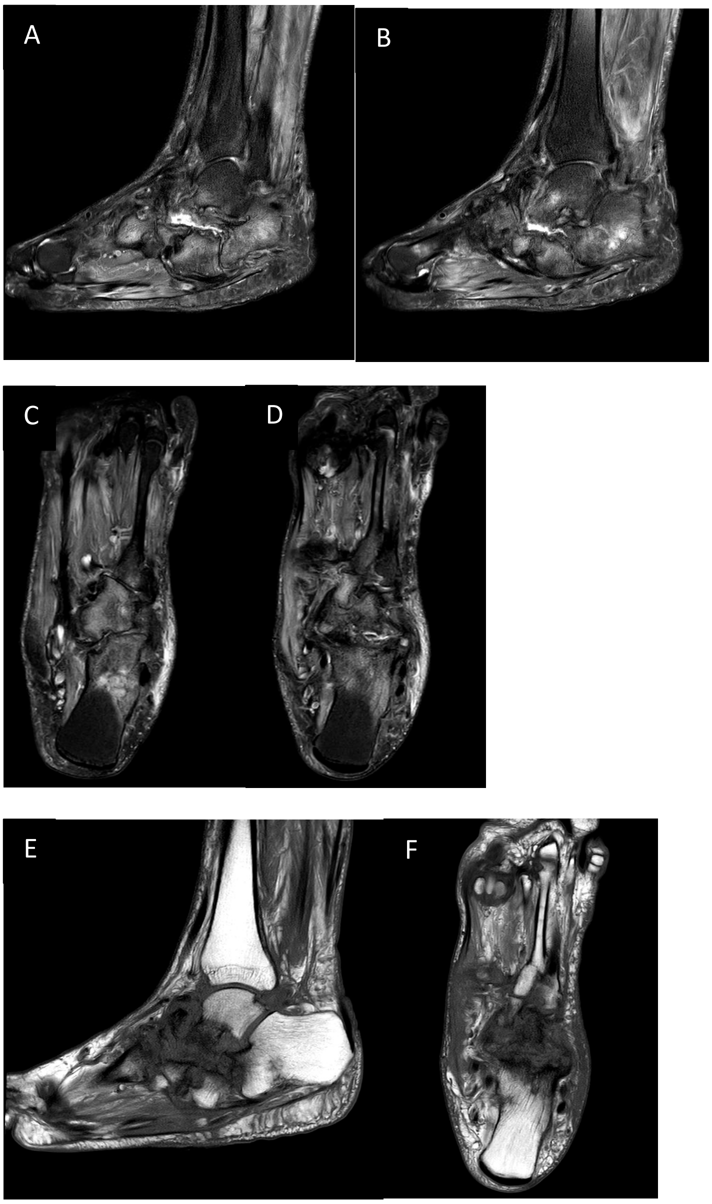
A
B
C
D
E
F

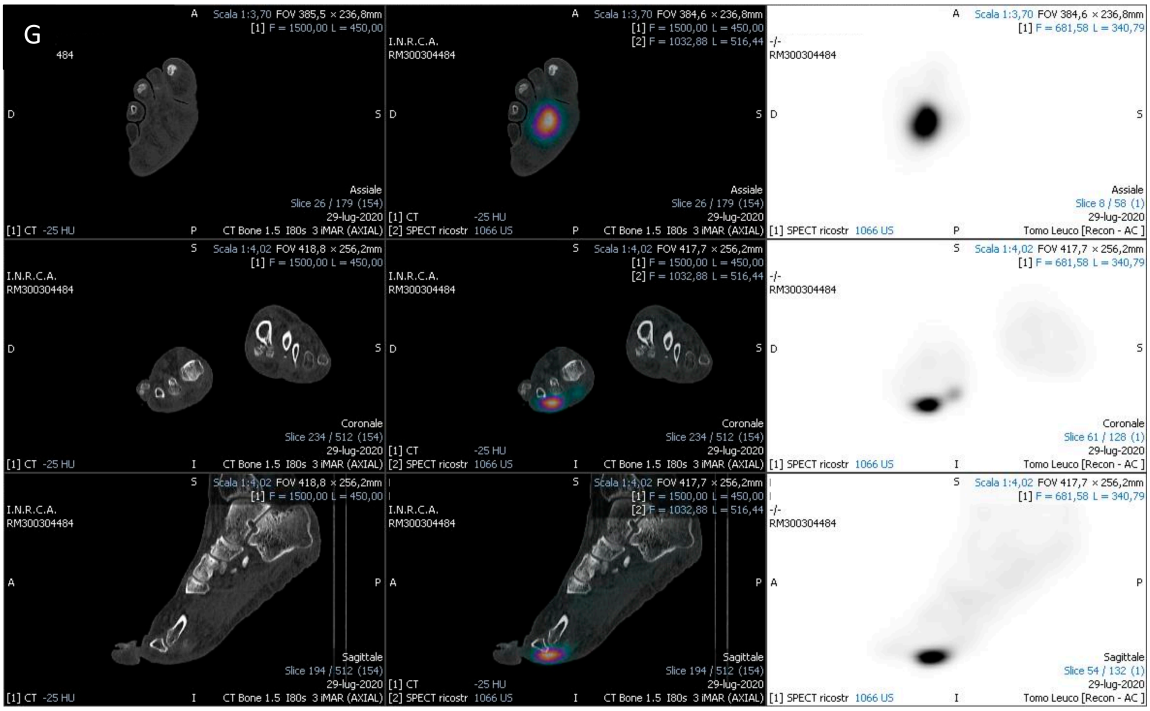

Supplementary Figure S3.

Case 3: a 55-year-old male with swollen, warm foot and ulceration on the sole of the foot

MRI: Short tau inversion recovery sequences (STIR) shows active Charcot foot (stage of fragmentation) with massive bone marrow edema in navicular bone, cuneiform, the talus and the calcaneus (A, B, C, D). T1 sequences show degree of bone destruction and fragmentation in the midfoot with huge signal drop in the fatty bone marrow, similar to signal drops in osteomyelitis (E, F); could be Charcot and a possible superimposed osteomyelitis.

HMPAO-WBC SPECT-CT demonstrates the presence of increased uptake in the soft tissue in the plantar aspect of the left foot but not in bone consistent with soft tissue infection (G).

Final diagnosis: Charcot foot.
