# Supplementary material for: Magnetic Resonance Imaging and 99Tc WBC-SPECT/CT Scanning in Differential Diagnosis between Osteomyelitis and Charcot Neuroarthropathy: A Case Series
Source: Tomography. 2024 Aug 22;10(8):1312–9. doi: 10.3390/tomography10080098 (PMC11359419; doi:10.3390/tomography10080098)
Supplement: Supplementary file 1 [file tomography-10-00098-s001.zip › Figure S4_Case 4.pdf]

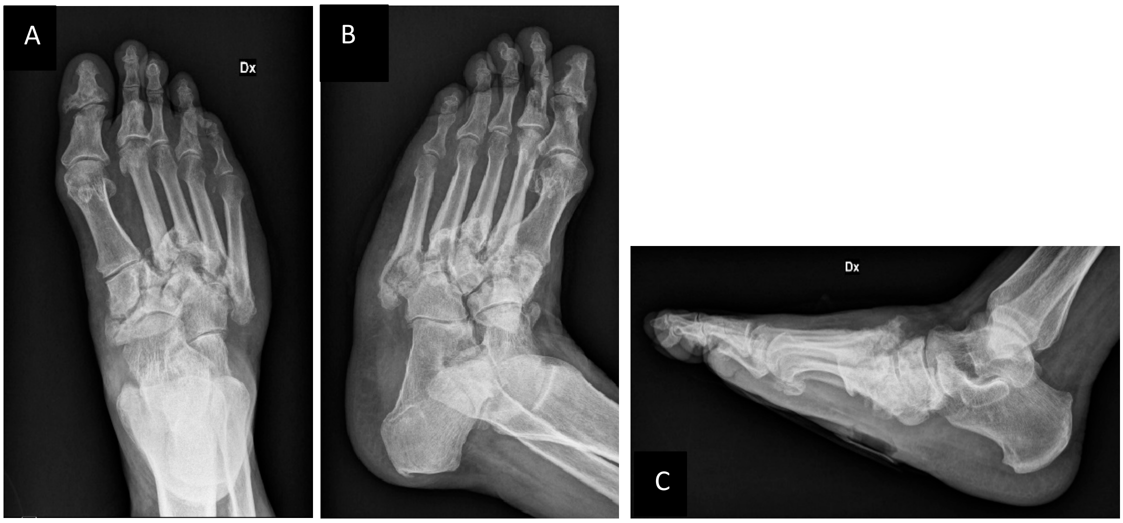
A
Dx
B
Dx
C

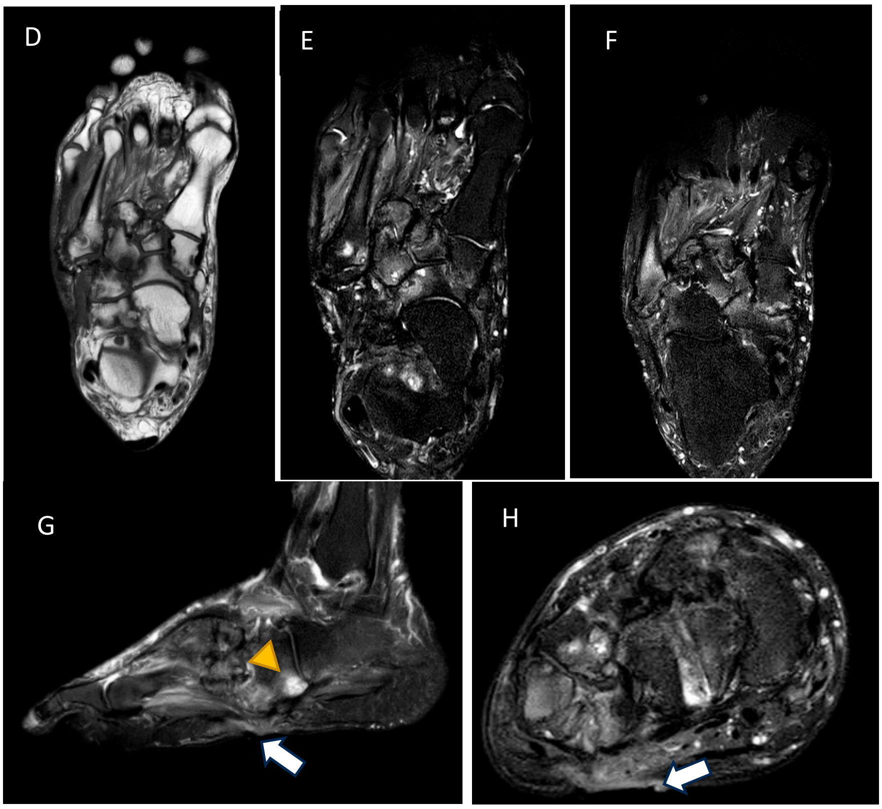
D
E
F
G
H

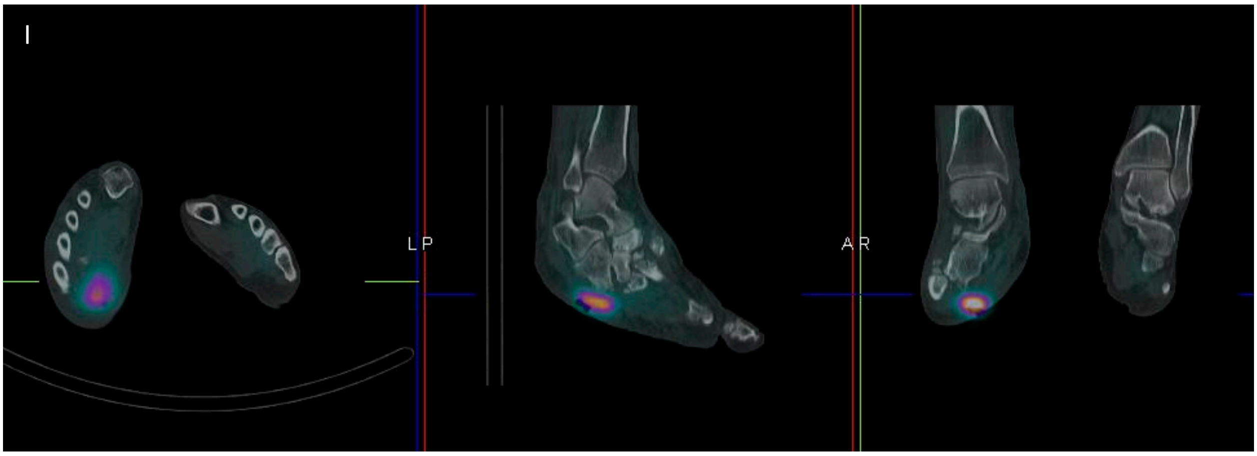

Supplementary Figure S4.

Case 4: a 56-year-old male patient with diabetes, polyneuropathy and a Charcot foot since 5 years. Now wound at right foot plantar side overlying mid-foot bony deformity.

X-ray showing midfoot fragmentation, destruction of the Lisfranc joint with sublussation and exuberant ossification (A-B-C)

MRI: T1 and STIR sequences showing bone marrow edema, erosion, disorganization and fragmentation with bone proliferation and debris formation at articular margins in the midfoot. (D-E-F). Sagittal and axial STIR sequences showing at the level of plantar wound a hyperintense signal in the cuboid bone and proximal part of 5th metatarsal with irregular aspect and fat induration (G-H). Could be possible osteomyelitis in reactivation of Chronic Charcot Arthropathy.

Tc-HMPAO-WBC SPECT-CT indicates an infectious process pertaining to the soft tissues of the middle third of the plantar vault, site of extensive ulcerative skin lesion, in the absence of signs of osteomyelitis (I).

Final diagnosis: Charcot without superimposed osteomyelitis.
