# Supplementary material for: Magnetic Resonance Imaging and 99Tc WBC-SPECT/CT Scanning in Differential Diagnosis between Osteomyelitis and Charcot Neuroarthropathy: A Case Series
Source: Tomography. 2024 Aug 22;10(8):1312–9. doi: 10.3390/tomography10080098 (PMC11359419; doi:10.3390/tomography10080098)
Supplement: Supplementary file 1 [file tomography-10-00098-s001.zip › Figure S5_Case 5.pdf]

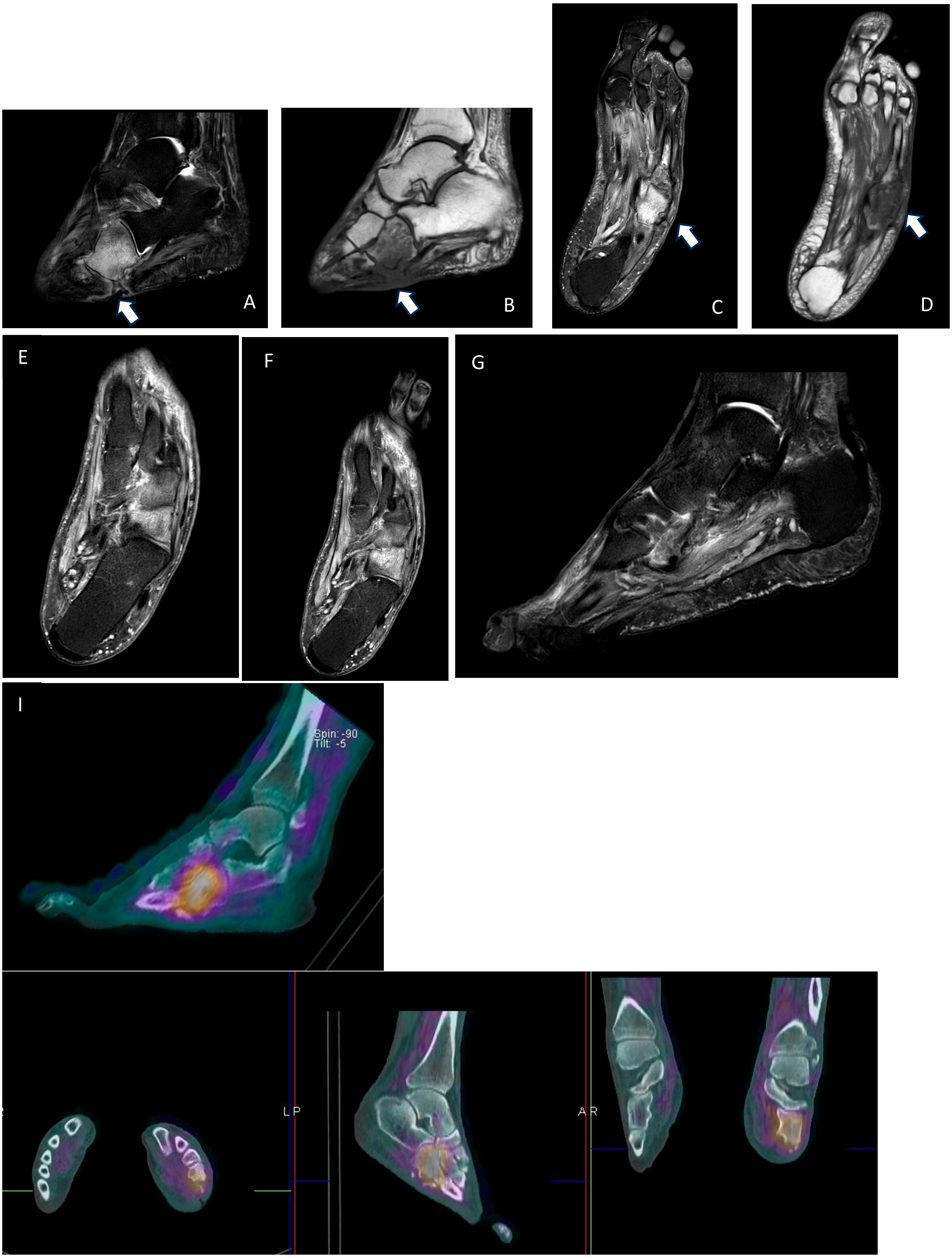
A
B
C
D
E
F
G
I
Spin: -90
Tilt: -5
LP
AR

Supplementary Figure S5.

Case 5: A case of a 62-year-old male patient with diabetes. He presented with an ulcer located on the site of previous amputation of the left fifth ray with swollen, warm foot and elevated temperature. The question from the referring clinician was if there was an osteomyelitis or a Charcot foot.

MRI: sagittal and coronal STIR and T1-weighted images showing diffuse bone marrow alteration within the cuboid bone, residual 5th metatarsal base and 4th metatarsal base with disappearance of bony contours and skin ulceration and sinus tract extending from the skin to the cuboid bone (A-B-C-D). Coronal and sagittal STIR showing classic bone marrow edema in the midfoot with soft tissue and muscle edema (E-F-G); could be active Charcot disease with superimposed osteomyelitis.

Tc-HMPAO-WBC SPECT-CT shows a pathological accumulation of marked leukocytes, in the cuboid bone, residual 5th metatarsal base and 4th metatarsal base with irregular cortex, bone fragmentation at the amputation side of metatarsal 5 and concomitant cutaneous fistulous tract in the lateral plantar area (I).

Final diagnosis: Charcot with superimposed osteomyelitis.
