# Supplementary material for: Magnetic Resonance Imaging and 99Tc WBC-SPECT/CT Scanning in Differential Diagnosis between Osteomyelitis and Charcot Neuroarthropathy: A Case Series
Source: Tomography. 2024 Aug 22;10(8):1312–9. doi: 10.3390/tomography10080098 (PMC11359419; doi:10.3390/tomography10080098)
Supplement: Supplementary file 1 [file tomography-10-00098-s001.zip › Figure S6_Case6.pdf]

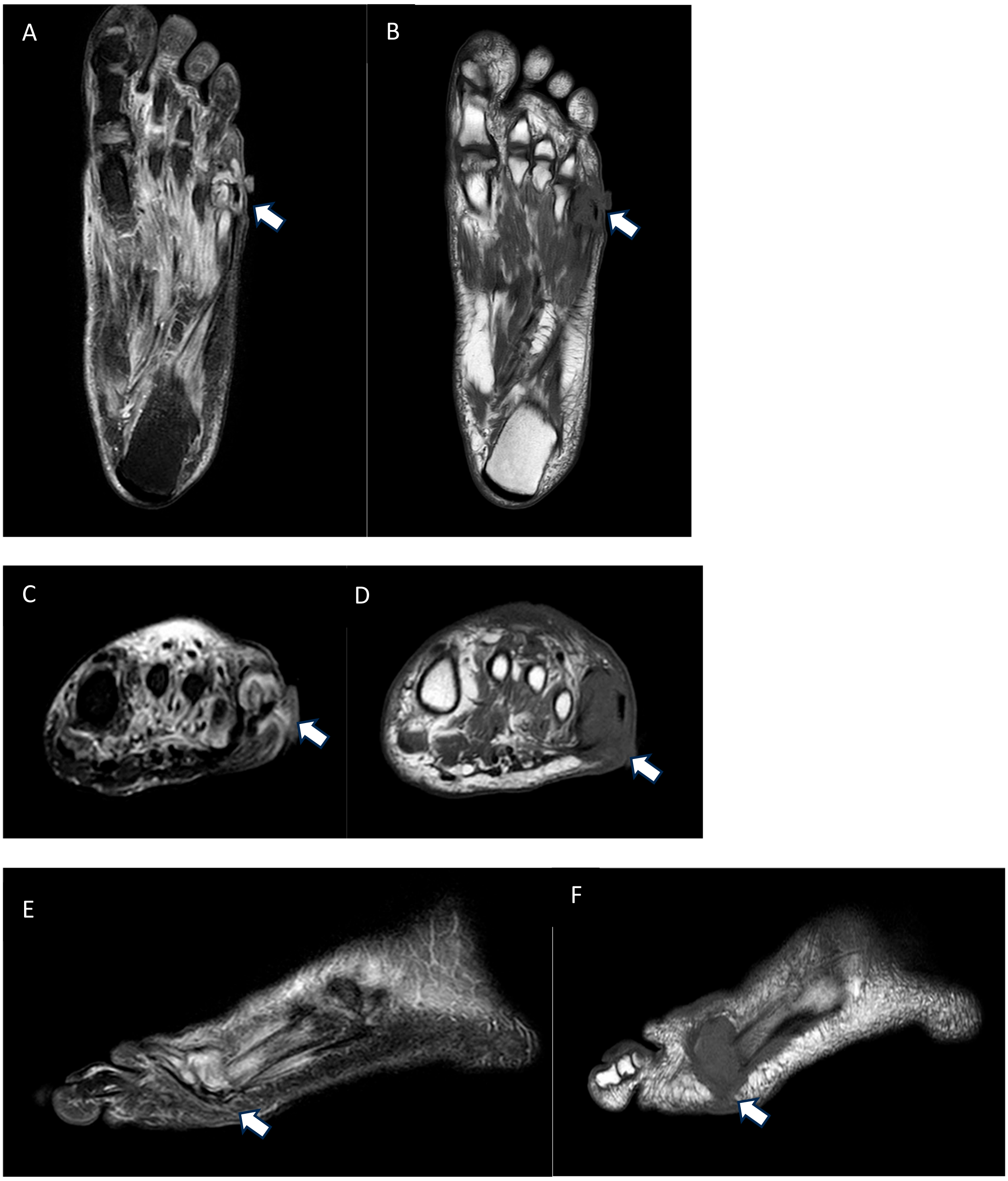
A
B
C
D
E
F

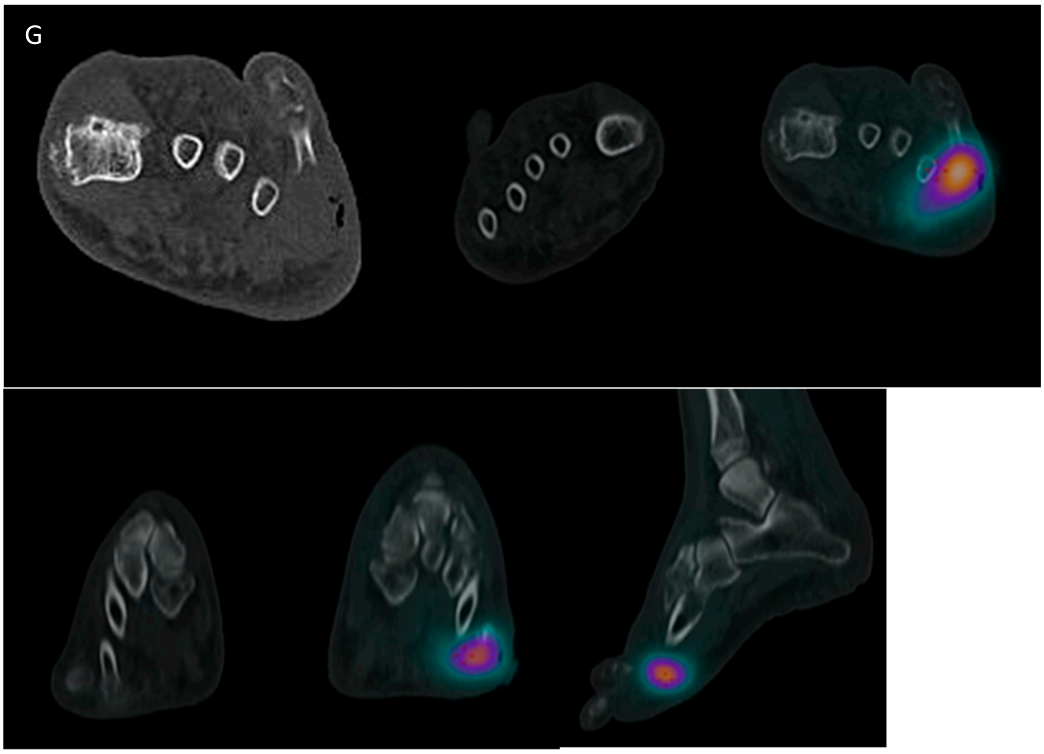

Supplementary Figure S6.

Case 6 : 57-year-old male with swollen, warm foot and ulceration on the lateral side of the midfoot, positive to the probe to bone test.

MRI: STIR and T1 sequences demonstrate skin ulceration and sinus tract extending from the skin to the 5th metatarsal head, showing a direct spread of infection. Diffuse bone marrow alteration is present within the 5th metatarsal head and proximal phalanx base with disappearance of bony contours in the sagittal T1-weighted image. There is no evidence of Charcot neuropathy on MRI (A-B-C-D-E-F).

99mTc-HMPAO-WBC SPECT-CT demonstrates the presence of WBC accumulation in the distal part of the 5th left metatarsus with structural abnormalities seen on the CT scan. Furthermore it shows an increased uptake in the soft tissue with inflammatory collection and sinus tract (G).

Final diagnosis: Osteomyelitis and Soft tissue infection.
