# Supplementary material for: Magnetic Resonance Imaging and 99Tc WBC-SPECT/CT Scanning in Differential Diagnosis between Osteomyelitis and Charcot Neuroarthropathy: A Case Series
Source: Tomography. 2024 Aug 22;10(8):1312–9. doi: 10.3390/tomography10080098 (PMC11359419; doi:10.3390/tomography10080098)
Supplement: Supplementary file 1 [file tomography-10-00098-s001.zip › Figure S7_Case7.pdf]

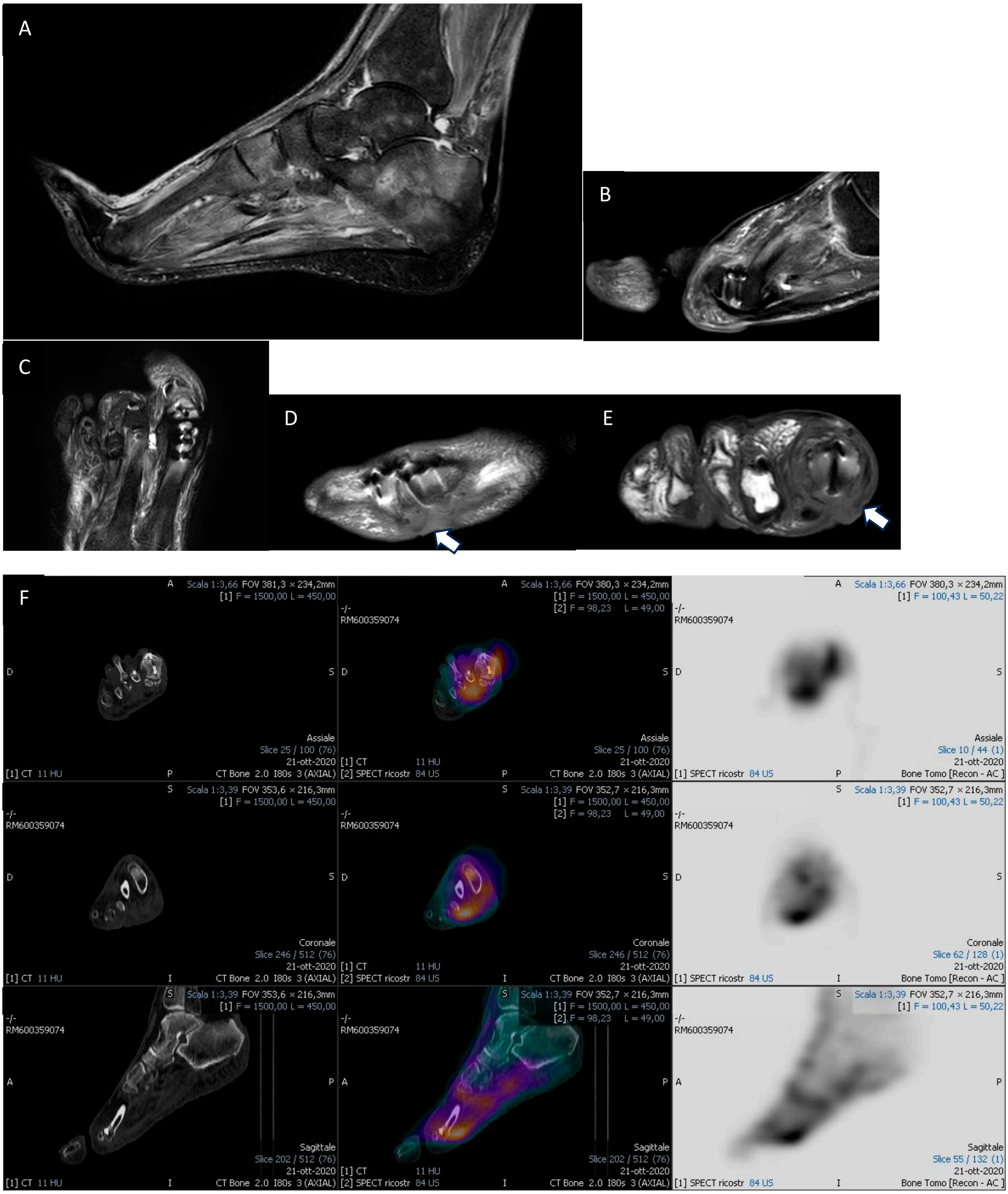


**Supplementary Figure S7.**

**Case 7**: 76-year-old female with erythematous, swollen foot and plantar ulceration at the level of the first metatarsophalangeal joint (MTPJ), where there is osteosynthesis material.

**MRI**: STIR sequence demonstrates early active Charcot foot: bone marrow edema in the midfoot and the soft tissue and muscle edema (A). The presence of osteosynthesis material at the site of interest (the first MTPJ) prevents proper evaluation of bone structures in this area due to artifacts from metallic fixation devices. It's only appreciable edematous thickening of the adjacent subcutaneous soft tissues (B, C, D, E).

**99mTc-HMPAO-WBC SPECT-CT** shows the presence of TC-WBC accumulation in the first MTPJ indicative of osteomyelitis

Final diagnosis: Charcot foot with superimposed osteomyelitis.
