# Supplementary material for: Magnetic Resonance Imaging and 99Tc WBC-SPECT/CT Scanning in Differential Diagnosis between Osteomyelitis and Charcot Neuroarthropathy: A Case Series
Source: Tomography. 2024 Aug 22;10(8):1312–9. doi: 10.3390/tomography10080098 (PMC11359419; doi:10.3390/tomography10080098)
Supplement: Supplementary file 1 [file tomography-10-00098-s001.zip › Figure S8_Case 8.pdf]

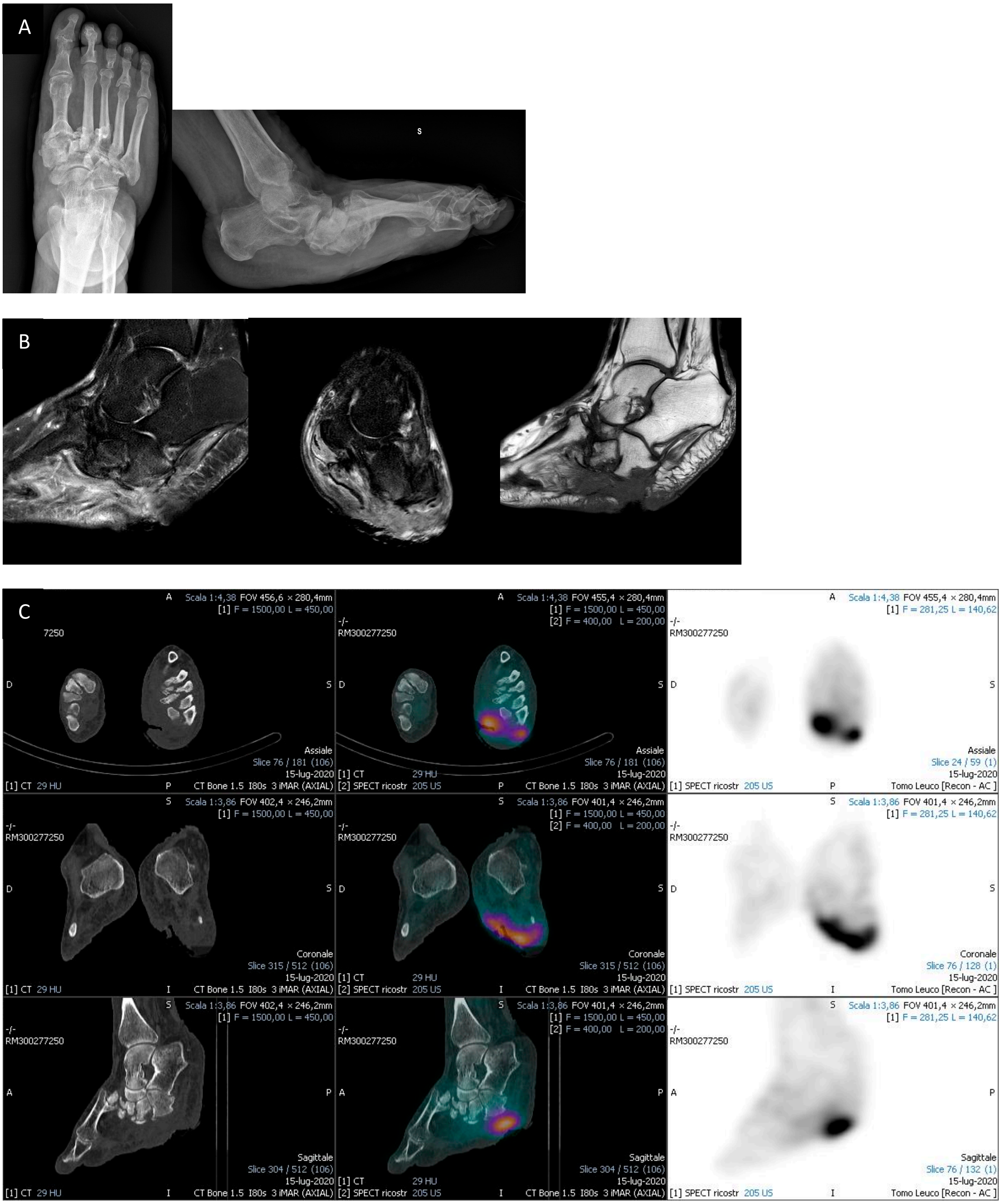
A
S
B
C
Scala 1:4,38 FOV 456,6 × 280,4mm
[1] F = 1500,00 L = 450,00
7250
D
S
A
P
Assiale
Slice 76 / 181 (106)
15-lug-2020
[1] CT 29 HU
CT Bone 1.5 I80s 3 iMAR (AXIAL)
Scala 1:4,38 FOV 455,4 × 280,4mm
[2] F = 400,00 L = 200,00
RM300277250
[2] SPECT ricostr 205 US
[1] F = 281,25 L = 140,62
Slice 24 / 59 (1)
[1] SPECT ricostr 205 US
Tomo Leuco [Recon - AC]
Scala 1:3,86 FOV 402,4 × 246,2mm
Scala 1:3,86 FOV 401,4 × 246,2mm
Coronale
Slice 315 / 512 (106)
Slice 76 / 128 (1)
I
Sagittale
Slice 304 / 512 (106)
Slice 76 / 132 (1)

Supplementary Figure S8.

Case 8: 64-year-old male with history of Charcot Arthropathy and rocker-bottom deformity presented with ulceration at the sole of the foot directly beneath the cuboid bone.

X-ray: classic features of late-stage Charcot foot with bone fragmentation and proliferation, rocker-bottom deformity (A)

MRI: STIR and T1 sequences demonstrate skin ulceration and probable sinus tract extending from the skin to the cuboid bone. There is a moderate amount of bone marrow edema within the cuboid bone without huge signal drop in the fatty bone marrow in the T1-weighted sequence and without disappearance of bony contours. It is a late-stage Charcot foot with uncertain underlying osteomyelitis (B).

99mTc-HMPAO-WBC SPECT-CT demonstrates increase in uptake in time located in the soft tissue directly beneath the cuboid bone, but not in bone, consistent with soft tissue infection (C).

Final diagnosis: Charcot foot without superimposed osteomyelitis.
