# Supplementary material for: Magnetic Resonance Imaging and 99Tc WBC-SPECT/CT Scanning in Differential Diagnosis between Osteomyelitis and Charcot Neuroarthropathy: A Case Series
Source: Tomography. 2024 Aug 22;10(8):1312–9. doi: 10.3390/tomography10080098 (PMC11359419; doi:10.3390/tomography10080098)
Supplement: Supplementary file 1 [file tomography-10-00098-s001.zip › Supplementary Table S1.pdf]

Case, age, sex	Location of ulcer	Clinical features	X-Ray findings	MRI findings	Location of MRI findings	Charcot foot	99Tc WBC-SPECT*	CT findings	CC-DFO	Outcome
1, 83 y, F	5th toe, L	Swollen, warm foot<br>Elevated temperature<br>Probe-to-bone positive	Loss of cortex<br>Radiolucency	BME<br>Cortical disruption	Proximal, middle and distal phalanx of 5th toe	NO	proximal and distal interphalangeal joints of 5th toe	Cortical disruption<br>Osteolysis	Definite	Wound healing<br>after antibiotic therapy
2, 44 y, F	lateral calcaneal site, L	Swollen, warm foot<br>Elevated temperature	Negative	BME (periarticular, subchondral distribution)	Calcaneus, navicular, medial and middle cuneiforms	YES<br>(Inflammation, Stage 0)	STI	Negative	Unlikely	Wound healing<br>after surgical debridement<br><br>Off-loading the foot<br>in an air cast walker
3, 55 y, M	plantar site, L	Swollen, warm foot<br>Elevated temperature	Midfoot fragmentation<br>Destruction (Lisfranc joint)<br>Sublussation	BME<br>Disorganization and fragmentation	Midfoot	YES<br>(Fragmentation phase)	STI	Negative	Unlikely	Wound healing<br>after surgical debridement<br><br>Off-loading the foot<br>in an air cast walker
4, 56 y, M	plantar site, R	Swollen, warm foot<br>Foot deformity<br>in history of Charcot Arthropathy	Midfoot fragmentation<br>Destruction (Lisfranc joint)<br>Sublussation<br>Exuberant ossification	BME<br>Disorganization and fragmentation<br>Debris formation at articular margins<br>Fusion of larger fragments	Midfoot and hindfoot	YES<br>(re-activation of Chronic Charcot foot)	STI	Disorganization, fragmentation of midfoot	Unlikely	Wound healing<br>after surgical debridement<br><br>Off-loading the foot<br>in an air cast walker
5, 62y, M	on the site of previous amputation of the 5th finger, L	Swollen, warm foot<br>Probe-to-bone positive<br>Erythrocyte sedimentation > 70 mm/h	Fragmentation of residual 5th metatarsal base<br>Loss of cortex, radiolucency (cuboid bone)	BME with cortical disruption<br>BME (periarticular, subcondral distribution)	Cuboid bone, residual base of the 5th metatarsal, 4th metatarsal base<br><br>midfoot	YES<br>(Inflammation, Stage 0)	Cuboid bone, residual 5th metatarsal base and 4th metatarsal base	Cortical disruption and subluxation	Definite	Wound healing<br>after antibiotic therapy<br><br>Off-loading the foot<br>in an air cast walker
6, 57y, M	5th metatarsal head, L	Swollen, warm foot<br>Probe-to-bone positive<br>Elevated temperature	Cortical disruption	BME with cortical disruption<br>+<br>Sinus tract in communication with subcutaneous fluid-collection	5th metatarsal head-neck and proximal phalanx base	NO	5th metatarsal head-neck and proximal phalanx base<br>+<br>STI<br>(infero-lateral margin of the 5th metatarsus)	Cortical disruption<br>+<br>Inflammatory collection in the adjacent soft tissues with a sinus tract	Definite	Amputation of the 5th ray and phlegmon drainage
7, 76y, F	plantar site, R	Swollen, warm foot<br>Probe-to-bone positive<br>Erythrocyte sedimentation > 70 mm/h	Osteosynthesis metallic material on the first metatarsophalangeal joint	BME (periarticular, subcondral distribution)	Midfoot, hindfoot and metatarsophalangeal joint	YES<br>(Inflammation, Stage 0)	Metatarsophalangeal joint	Negative	Probable	Wound healing<br>after antibiotic therapy<br><br>Off-loading the foot<br>in an air cast walker

8, 64y, M	plantar site, L	Swollen , warm foot Foot deformity in history of Charcot Arthropathy	Midfoot fragmentation Destruction (Lisfranc joint), Sublussation Exuberant ossification	BME Disorganization and fragmentation Debris formation at articular margins Fusion of larger fragments	Midfoot and hindfoot	YES (re-activation of Chronic Charcot foot)	STI (left plantar midfoot)	Disorganization and fragmentation of midfoot and hindfoot	Unlikely	Wound healing after surgical debridement<br><br>Off-loading the foot in an air cast walker

***Areas of Increased Uptake**

F: female; M: male; L: left; R: right; BME: Bone Marrow Edema; STI: Soft Tissue Infection.
